# Transcriptomic differences in MSA clinical variants

**DOI:** 10.1038/s41598-020-66221-4

**Published:** 2020-06-25

**Authors:** Alexandra Pérez-Soriano, Magdalena Arnal Segura, Teresa Botta-Orfila, Darly Giraldo, Manel Fernández, Yaroslau Compta, Rubén Fernández-Santiago, Mario Ezquerra, Gian G. Tartaglia, M. J. Martí, the Catalan MSA Registry (CMSAR), Esteban Muñoz, Javier Pagonabarraga, Francesc Valldeoriola, Jorge Hernández-Vara, Serge Jauma Classen, Victor Puente, Claustre Pont, Núria Caballol, Oriol de Fàbregues, Asunción Ávila, Matilde Calopa, Carles Gaig, Pau Pastor, Montserrat Pujol, Joan Santamaria, Lluís Planellás, Ana Cámara

**Affiliations:** 1 Parkinson’s Disease & Movement Disorders Unit, Hospital Clínic/IDIBAPS/CIBERNED/European Reference Network for Rare Neurological Diseases (ERN-RND)/Institut de Neurociències, University of Barcelona, Catalonia, Spain; 2 Laboratory of Parkinson Disease and Other Neurodegenerative Movement Disorders, IDIBAPS, Barcelona, Catalonia Spain; 3 Gene Function and Evolution Group, Centre for Genomic Regulation (CRG), Parc de Recerca Biomédica de Barcelona (PRBB), Barcelona, Catalonia Spain; 4 Human Computational Biology Group, Hospital del Mar Medical Research Institute (IMIM), Parc de Recerca Biomédica de Barcelona (PRBB), Barcelona, Catalonia Spain; 5 Biological Fluids Biobank; IDIBAPS-Hospital Clinic of Barcelona, Barcelona, Catalonia Spain; 6 María de Maeztu Unit of Excellence (Institute of Neurosciences, University of Barcelona), Ministry of Science, Innovation and Universities, Barcelona, Catalonia Spain; 7 Institució Catalana de Recerca I Estudis Avançats (ICREA), Universitat Pompeu Fabra (UPF), Barcelona, Spain; 8 Movement Disorders Unit, Hospital de Sant Pau, Barcelona, Catalonia Spain; 9 Movement Disorders Unit, Hospital Vall d’Hebron, Barcelona, Catalonia Spain; 10 Movement Disorders Unit, Hospital de Bellvitge, Barcelona, Catalonia Spain; 11 Movement Disorders Unit, Hospital del Mar, Barcelona, Catalonia Spain; 12 Movement Disorders Specialist, Hospital General de Granollers, Granollers, Catalonia Spain; 13 Movement Disorders Unit, Hospital de Sant Joan Despi, Moisès Broggi-Consorci Sanitari integra, L’Hospitalet de Llobregat, Catalonia, Spain; 14 Movement Disorders Unit, Centre Mèdic Teknon, Barcelona, Catalonia Spain; 15 Movement Disorders Unit, Department of Neurology, Hospital Universitari Mutua de Terrassa, Terrassa, Catalonia Spain; 16 Movement Disorders Specialist, Hospital Santa María de Lleída, Lleída, Catalonia Spain

**Keywords:** Neurological disorders, Movement disorders, Computational biology and bioinformatics, Neuroscience, Neurology

## Abstract

Background: Multiple system atrophy (MSA) is a rare oligodendroglial synucleinopathy of unknown etiopathogenesis including two major clinical variants with predominant parkinsonism (MSA-P) or cerebellar dysfunction (MSA-C). Objective: To identify novel disease mechanisms we performed a blood transcriptomic study investigating differential gene expression changes and biological process alterations in MSA and its clinical subtypes. Methods: We compared the transcriptome from rigorously gender and age-balanced groups of 10 probable MSA-P, 10 probable MSA-C cases, 10 controls from the Catalan MSA Registry (CMSAR), and 10 Parkinson Disease (PD) patients. Results: Gene set enrichment analyses showed prominent positive enrichment in processes related to immunity and inflammation in all groups, and a negative enrichment in cell differentiation and development of the nervous system in both MSA-P and PD, in contrast to protein translation and processing in MSA-C. Gene set enrichment analysis using expression patterns in different brain regions as a reference also showed distinct results between the different synucleinopathies. Conclusions: In line with the two major phenotypes described in the clinic, our data suggest that gene expression and biological processes might be differentially affected in MSA-P and MSA-C. Future studies using larger sample sizes are warranted to confirm these results.

## Introduction

Multiple system atrophy (MSA) is an adult-onset neurodegenerative disease characterized by autonomic failure and an early motor predominance of cerebellar symptoms such as ataxia (MSA-C) or a poor levodopa responsive parkinsonism (MSA-P)^1^. Pathologically, MSA can encompass predominant olivopontocerebellar atrophy or striatonigral degeneration with neuronal loss, gliosis and glial cytoplasmatic inclusions (GCI) mainly containing aggregated α-synuclein (SNCA), among other proteins. The two pathological distributions correlate with the clinical phenotype, although in late stages most cases have mixed clinicopathological profiles^2^. Clinical consensus guidelines are key for diagnosis allowing for an up to probable diagnosis; however definite diagnosis requires neuropathological confirmation^3^. Although following consensus criteria improves diagnostic accuracy significantly^4,5^, in the clinic about 20^6^ to 40%^7^ of patients are still misdiagnosed.

Etiologically, MSA is a rare and sporadic disease with few recognized monogenic causes^8^. Association of genetic risk polymorphisms in the *SNCA*^9^ and the *MAPT*^10^ genes among others have been reported^11–13^, but results have not always been replicated. *SNCA* mRNA or protein expression level studies in specific brain regions have also been controversial^14–16^. More recently, transcriptional pathways regulating gene expression have been explored in MSA brains^17,18^, identifying differentially expressed candidate genes potentially involved in the pathological cascade of MSA^19^. A limitation to most studies is linked to the clinical heterogeneity of MSA, which hampers selection of homogenous cohorts of patients. To tackle this problem, we built a large multicenter Catalan MSA clinical registry and bio-repository (CMSAR). Using peripheral blood from clinically well-characterized and carefully balanced MSA cases by age, gender, and phenotype, we explored potential transcriptomic alterations occurring in MSA, or its subtypes MSA-P and MSA-C, comparatively with healthy controls or Parkinson disease (PD) cases. Our aim was to identify differential gene expression patterns associated with MSA, which may unveil distinct biological and molecular pathways involved in disease pathogenesis.

## Results

We first used a resampling strategy to identify differentially expressed transcript cluster IDs (DE TCI) corresponding to the genes most recurrently showing differential expression changes across iterations. We found an enrichment of transcripts with low scores in the distribution of q-values obtained with the Kruskal-Wallis test for the transcripts detected with a p-value <0.05 at least one time in the resampling iterations (Fig. S1). The left side histogram peak for each contrast corresponds to transcripts showing expression differences not attributable to random effects. Moreover, we found that Kruskal-Wallis q-values obtained by resampling do correlate with the number of times a gene is differentially expressed across the multiple iterations. Therefore, the most recurrent DE TCI are also those showing the lower q-values (Fig. S2). More specifically, we used a cut-off of Kruskal-Wallis q-value < 0.0001 to detect the most significant DE TCI (Table S1). Altogether, these findings indicate that the biological expression differences occurring among groups statistically are not due to random effects.

**Figure 1:**
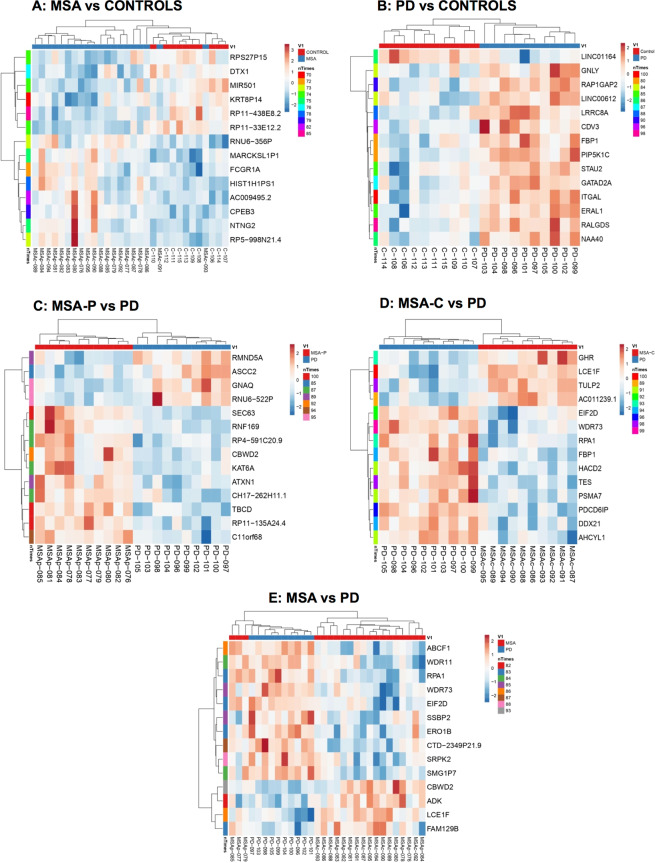
Resampling analysis heatmap representations. Heatmap representation of differentially expressed transcript cluster ID (DE TCI) detected across comparisons by resampling analysis (RA) for contrasts MSA vs. Controls (**A**), PD vs. Controls (**B**), MSA-P vs. PD (**C**), MSA-C vs. PD (**D**), MSA vs. PD (**E**). N indicates number of iterations a gene was found to be differentially expressed out of 100 iterations. Unsupervised tree ordering for clusters is given by tightest clusters first, with both rows and columns clustered using correlation distance and average linkage. Red shading indicates up-regulation and blue shading indicates down-regulation. Note that when analyzing by clinical phenotype heatmap discrimination improves. PD = Parkinson disease, MSA = Multiple system atrophy (P = Parkinsonian phenotype, C = Cerebellar phenotype).

Subsequently, comparing MSA and healthy controls, the resampling analysis revealed 28 DE TCI in at least 70 of 100 iterations (Fig. 1A), being 7 of these protein coding genes, of which 3 were involved in nervous system development (*CPEB3*, *DTX1* and *NTNG2*). We subsequently stratified MSA cases by disease phenotype and observed a higher amount of DE TCI in MSA-P, with 113 DE TCI in 70 of 100 iterations as compared to controls, of which 51 were protein coding genes. However, in MSA-C we only found 8 DE TCI in 70 of 100 iterations encompassing only two protein coding genes (*PLEKHG1* and *C1orf56*) (Table S2). Comparatively, expression changes in PD vs. controls were larger and more specific than in MSA vs. controls, revealing 26 DE TCI in 80 of 100 iterations (Fig. 1B), 12 of which were protein coding DE TCI genes related to cell adhesion, vesicle trafficking, and metabolism. Per subtype, MSA-P vs. PD resulted in 21 DE TCI in 80 of 100 iterations, showing 14 protein coding transcripts (Fig. 1C), which were mostly related to cytoskeleton, protein modification, and development. In MSA-C vs. PD we found the largest amount of differences with 57 DE TCI detected in 80 of 100 iterations, which included 36 protein coding genes (Fig. 1D). Of note, 12 of these were RNA binding proteins, 6 of which were detected in 85 of 100 iterations (*TES*, *DDX21*, *EIF2D*, *AHCYL1*, *SNUPN*, *ADK*). Altogether these results indicate a potentially heterogeneous expression profile in MSA (Fig. 1E), possibly due to specific differences dependent on the clinical phenotype (MSA-P or MSA-C), with an apparent higher degree of heterogeneity in MSA-C.

**Figure 2:**
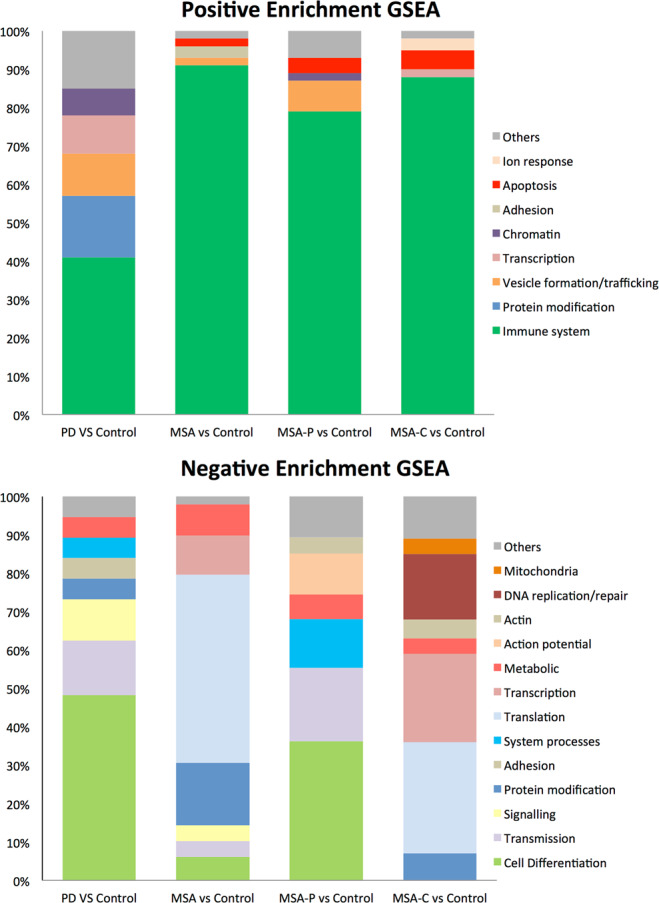
Gene set enrichment analysis (GSEA). Upper and lower bar graphs represent positive and negative enrichment results for each contrast respectively. Y axis indicates percentage of gene sets related to a specific biological process group depicted with a specific color seen in legend. Results show top 100 sets when applicable ordered by nominal enrichment score selected using cut-off P-value below 0.05.

**Figure 3:**
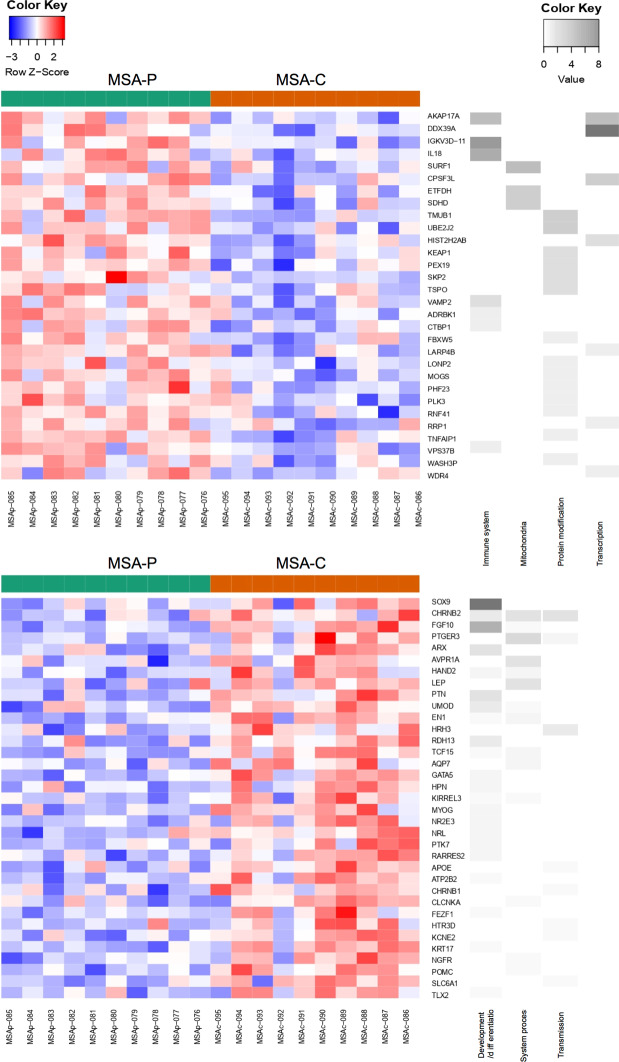
GSEA analysis in MSA-P vs. MSA-C. Positive enrichment GSEA results comparing MSA-P vs. MSA-C on top and negative enrichment below, represented as heatmaps where green indicates MSA-P cases and orange indicates MSA-C cases, red shading represents up-regulation and blue shading down-regulation. Grey scale heatmap to the left of color heatmap indicates each gene’s corresponding biological processes. Only genes that were related to these specific biological processes have been selected with a cut-off p-value < 0.01. MSA = Multiple system atrophy (P = Parkinsonian phenotype, C = Cerebellar phenotype).

**Figure 4:**
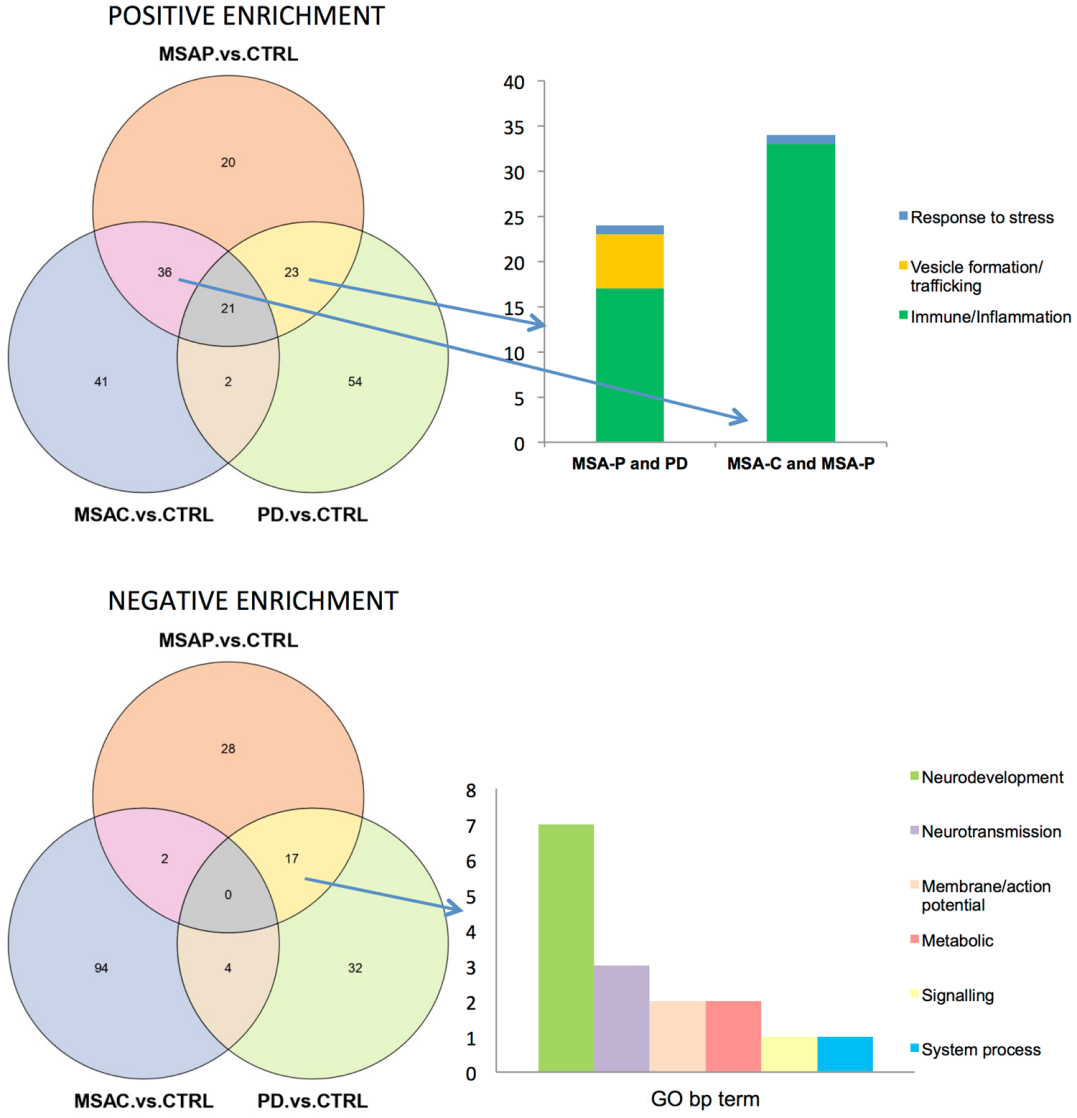
Common GSEA results between both MSA variants and PD. Venn diagrams representing common results of the top 100 gene sets ordered by NES score and selected using a p-value below 0.05. Left bar graph represents the number of gene sets related to a biological process with overlapping results between positive enrichment gene sets of MSA-P and PD cases compared to controls and MSA-P and MSA-C compared to controls and negative enrichment gene sets of MSA-P and PD cases compared to controls. NES = Nominal enrichment score, CTRL = control, PD = Parkinson disease, MSA = Multiple system atrophy (P = Parkinsonian phenotype, C = Cerebellar phenotype), GO = gene ontology, bp= biological process, DE = differentially expressed, PE = positive enrichment, NE = Negative enrichment.

To explore whether the expression differences in MSA or its subtypes could impact specific biological functions we performed a gene set enrichment analysis (GSEA). Regarding positive enrichment (Fig. 2) in the overall MSA group, as well as when stratifying by subtype, we consistently observed processes related to the immune system and inflammation among the top 100 significantly enriched gene sets. As a control experiment, we did the same analysis using balanced subgroups of controls and did not observe such an enrichment, thus indicating a specific effect of MSA on immunity. In PD, beyond immunity, positive enrichment was related to transcription, protein modification, and vesicle trafficking. Regarding negative enrichment (Fig. 2), both MSA-P and PD groups revealed gene sets mainly related to cellular differentiation and development of the nervous system. Conversely, negative enrichment in the overall MSA and MSA-C groups were mostly related to protein translation and protein modification processes. In general, we found that MSA-P and MSA-C seem to have different enriched GO biological pathway gene sets (Fig. 3), and while MSA-P cases share common gene sets with both PD and MSA-C, MSA-C barely shows overlapping results with PD cases (Fig. 4). Of note, when assessing all MSA cases vs. PD cases we found a significant positive enrichment for biological processes related to the autonomic system such as regulation of blood pressure regulation, urine volume, water loss via skin, heat generation and vasoconstriction. GSEA data for all contrasts is presented in Sfile1.

**Figure 5:**
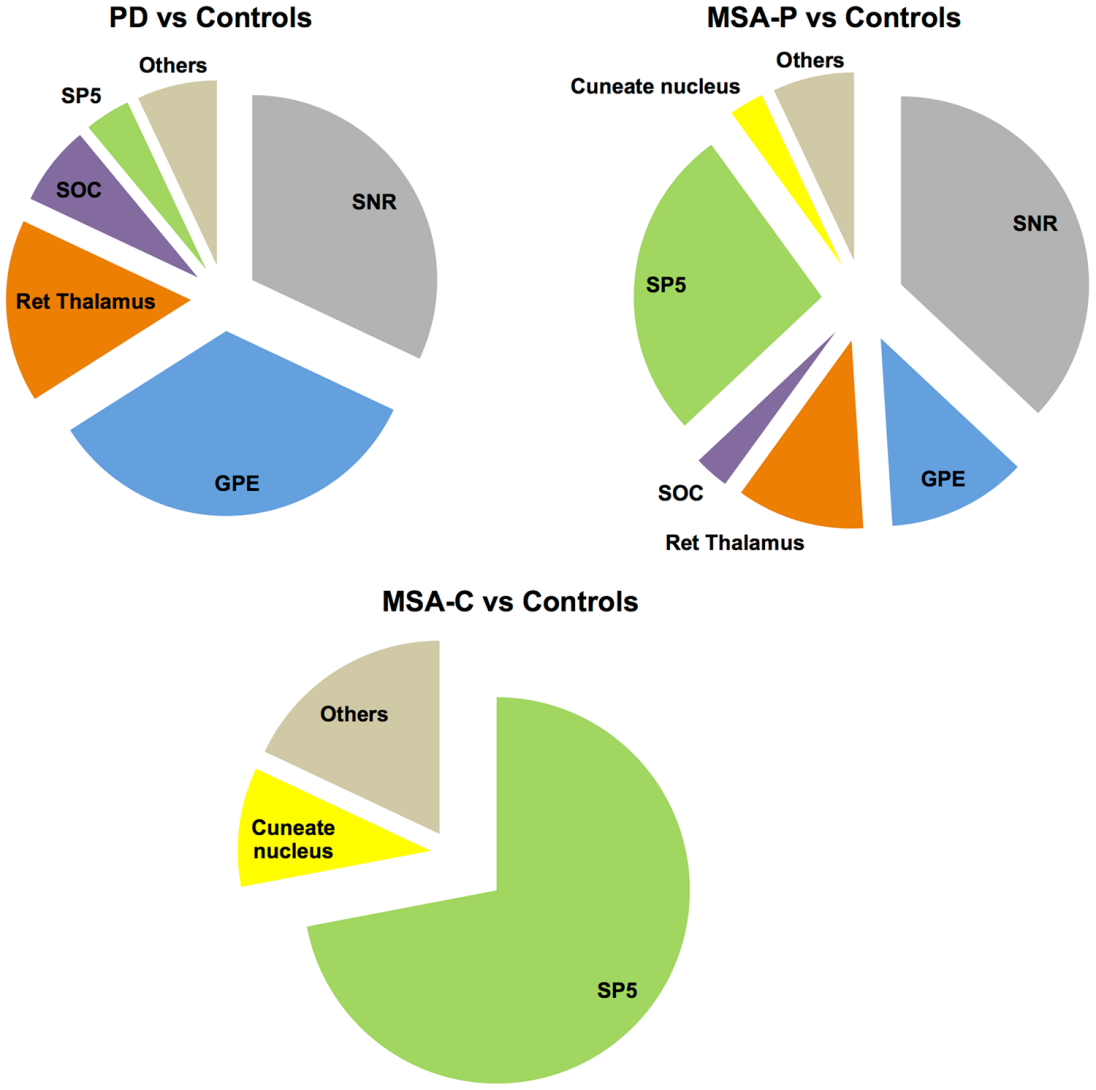
Analysis of DE TCI in MSA in a brain regions context. Pie charts showing proportion of times a brain region was positively enriched in comparison to another brain region in PD vs. Controls, MSA-P vs. Controls, and MSA-C vs. Controls. Note that MSA- P cases show similar regions to PD and region SP5 is significantly enriched in both MSA subtypes. PD = Parkinson disease, MSA = Multiple system atrophy (P = Parkinsonian phenotype, C = Cerebellar phenotype), SP5 = Spinal trigeminal area.

To investigate whether the DE TCI we identified in blood could also be expressed in the human brain we overlapped our results with Allen’s Brain atlas which is a comprehensive transcriptomic reference atlas for different brain regions. Doing so, we again found a distinctive gene expression pattern in MSA and in PD. In PD, compared to controls or to MSA cases, positively enriched gene sets included transcripts which are highly expressed in substantia nigra pars reticulata (SNr), globus pallidus externus (GPE), and reticular thalamus in comparison to other brain regions. On the contrary, enriched gene sets in MSA and predominantly in MSA-C cases corresponded to genes highly expressed in the spinal trigeminal nucleus (SP5). This analysis again showed a higher degree of similarities between MSA-P and PD as compared to MSA-C (Fig. 5).

## Discussion

In this study for the first time we report transcriptomic changes occurring in peripheral blood from MSA subjects taking into account clinical subtypes and comparing them to healthy controls and to another α-synucleinopathy such as PD. In keeping with previous literature^20^, we found a lower number of significant gene expression differences in MSA than in PD when compared to controls. This observation may be related at least in part, to the clinical heterogeneity of the MSA group as a whole since two distinct clinicopathological phenotypes are well-described for this disease^1^. Indeed, after stratifying by subtype we found a larger amount of expression changes, specifically in MSA-P cases, suggesting a possible differential transcriptomic regulation underlining the two main MSA phenotypes. Moreover, we also found that the expression profile in MSA-P seemed to share more gene sets with PD than with MSA-C, possibly in relation to clinical similarities. In line with our findings, a recent microRNA study^21^ has also reported differential deregulation of microRNAs between the two MSA subtypes.

Previous transcriptomic studies in MSA were assessed in brain tissue. The first brain MSA study using RNA sequencing, compared gene expression between white and grey matter regions^17^. This study reported differential tissue-specific transcriptional changes with a down-regulation of inflammatory processes in MSA white matter along with an unbalanced iron homeostasis in grey matter. Although it is difficult to directly compare our results to brain studies, we also found that gene expression changes detected in MSA blood affected immunity and inflammation regardless the clinical subtype. These were mostly related to innate immunity, especially to cytokine response and inflammation, including deregulation of interferon, IL1, IL6, and IL8 gene sets. Accordingly, it is important to mention that brain region specific neuroinflammation has been shown to be key in the pathogenesis of MSA, where increased levels of pro-inflammatory cytokines, as well as microglial activation by aggregated α-synuclein are well-described in the disease^22,23^. Conversely, PD cases showed enrichment not only in immunity processes, but also in transcriptional and protein modification processes. Per contrary, negative enrichment was comparatively more heterogeneous affecting cell differentiation and neural development in MSA-P (similar to PD), and protein translation and processing in MSA-C. These findings suggest that both MSA-P and MSA-C may involve different biological processes. Notably, the whole MSA group showed an upregulation of processes related to the autonomic system when compared to PD. Although both diseases share autonomic dysfunction, MSA cases typically have a more severe autonomic dysfunction than PD cases, which would be in line with this finding.

Although gene expression is tissue-specific^17^ it has been reported that changes in blood may reflect ongoing molecular changes in the brain^24,25^. When overlapping our results to Allen’s brain atlas we found that differentially expressed  genes in PD and MSA-P revealed an overrepresentation of genes involved in the nigrostriatal pathway suggesting a possible biological significance to our findings. Genes related to these regions were involved with immune response, cell migration and developmental processes. In contrast, the spinal trigeminal nuclei (SP5), has not been previously related to MSA, however its neighboring areas such as the middle cerebellar peduncle and pontine reticular formation are typically affected by myelin degeneration and by presence of GCI^26^. Gene sets that were overexpressed in our cohort in this region were mostly associated with interferon response, which is mediated by interferon-gamma receptors present in the caudal part of the spinal trigeminal nuclei. These neurons have been postulated to modulate infection or antigen related responses^27^. Yet, caution must be taken in extrapolating our findings in blood to brain.

Our study has limitations. First, blood can be influenced by disease extrinsic causes, thus we aimed for age and gender homogeneous groups, excluding cases with significant comorbidities such as diabetes mellitus and including cases with a probable MSA diagnostic certainty. Second, disease heterogeneity is large, in part due to the two distinct clinicopathological phenotypes; therefore we equally stratified analyses by disease variant. Third, most MSA cases were in moderate to late stages of the disease, as happens in most cohorts that only assess cases with probably certainty^28^ however since MSA progresses rapidly, small differences in disease duration or stage may impact the affected biological processes. Future studies should involve early stage cases as well to see if expression changes significantly differ at the beginning of the disease . Fourth, the sample size was limited, which is usually the case when studying rare diseases such as MSA. Still, our results may help for the design of future MSA studies, ideally by stimulating joint international collaborative efforts, which would allow for larger MSA studies leading towards more homogenous and clinically well-characterized MSA samples, carefully stratified by MSA clinical variants and disease stages. Such studies can expand and validate our findings, whilst further contributing to elucidating the underlying molecular pathogenesis of MSA.

In summary, our gene set enrichment analysis revealed positive enrichment of biological processes mostly related to immunity and inflammation but differed depending on the phenotypic variant when assessing negative enrichment, suggesting that the clinical variant should be taken into account in MSA studies. Future studies using larger homogeneous samples classifying by clinical subtype and also by disease stage are needed to validate our results.

## Methods

### Sample collection

The study conformed to the principles of the Declaration of Helsinki and the Belmont Report. All participants gave written informed consent, and the study was approved by the ethics committee from the Hospital Clínic  de Barcelona. Personal data and subject samples were codified to preserve confidentiality. The study included 20 probable MSA subjects from the CMSAR (10 MSA-P and 10 MSA-C cases), 10 healthy controls and 10 PD cases. MSA variants were diagnosed by an experienced movement disorder specialist, and phenotype was assigned depending on predominant motor symptom at disease onset following clinical consensus criteria^3^. All subjects were gender and age-matched between groups (Table S3). Subjects with diabetes mellitus were excluded in all groups. Healthy controls had no history of neurological diseases or any other serious illnesses. Blood extraction and RNA isolation were done using consensus guidelines from the Parkinson’s Progression Markers Initiative (PPMI) (https://www.ppmi-info.org). Briefly, 2.5 ml of blood in fasting was collected in a PAXgene tube, incubated overnight at room temperature, and stored at −80 °C until use. Prior to experiment, RNA isolation was done simultaneously in all samples following the PAXgene Blood RNA Kit (QIAGEN) manual protocol^29^. RNA concentration was determined on a NanoDrop ND-3300 Fluorospectrometer (Thermo Fisher Sci.). Quality control was performed using a Bioanalyzer instrument (Agilent).

### Microarray hybridization

Analyses using the commercially available array Clariom D Assay (Thermo Fisher Sci.) were done at the High Technology Unit (UAT) at Vall d’Hebron Research Institut (VHIR), with a GeneChip System 3000 (Affymetrix, Thermo Fisher Sci). A concentration of 100 ng of total RNA from each sample was used as starting material. The quality of the isolated RNA was previously measured by capillary electrophoresis (Bioanalyzer 2100, Agilent). Briefly, single stranded cDNA suitable for labeling was generated from total RNA using the GeneChip WT Plus Reagent Kit (Thermo Fisher Sci) according to the manufacturer’s instructions. Purified sense-strand cDNA was fragmented, labeled and hybridized to the arrays using the GeneChip WT Plus Terminal Labeling and Hybridization Kit from the same manufacturer. After array scanning, raw data quality control was performed to check the performance of the whole processing.

### Resampling analysis

We performed a resampling analysis using gender balanced subgroups of 4 samples in each condition. A total of 100 analyses of differentially expressed transcription cluster IDs (DE TCI) using the “limma” method^30^ implemented in R were performed with different subgroups of samples for each comparison avoiding repetitions. In order to obtain a list with the most statistically significant DE TCI in the resampling analysis and avoid the false positives generated in multiple comparison approaches, a resampling analysis was performed again with class label permutation generating 100 random contrasts with balanced subgroups from the whole dataset. We applied a Kruskal-Wallis test comparing the p-value distribution for each gene obtained for each contrast against the random p-value distribution generated in the resampling analysis with class label permutation in the 100 iterations. p-values obtained in the Kruskal-Wallis test for each transcript were converted to q-values being a more accurate statistic to control for the False Discovery Rate (FDR). The distribution of q-values obtained in the Kruskal-Wallis test for DE TCI with a p-value < 0.05 at least one time in the resampling analysis of 100 iterations is shown in the supplement (Fig. S1). An enrichment of DE TCI is observed on the histogram’s left peak for all contrasts. Pearson correlations were used to demonstrate that Kruskal-Wallis q-values are correlated with the number of times a transcript is DE in the resampling analysis (Fig. S2). A cut-off of Kruskal-Wallis q-value < 0.0001 was applied to identify the lists of most significant DE TCI (Table S1). The number of times a TCI was found as differentially expressed (DE) with a p-value < 0.05 and the sum of logFC among the 100 iterations was used to rank transcripts based on their recurrence and consistency.

### Gene set enrichment analysis (GSEA)

GSEA^31^ was performed in order to retrieve functional pathways with the gene set collection ‘GO biological process C5bp available at the Molecular Signatures Database (MSigDB)^32^. A Pre-ranked analysis was performed ranking the genes using a score based on the sum of logFC among the 100 iterations in the resampling analysis.

### Allen brain atlas gene expression comparison

Microarray expression data was downloaded from the Allen Brain Atlas (http://human.brain-map.org/static/download). Microarray data pre-processing and normalization details can be found online (http://help.brain-map.org/display/humanbrain/Documentation). Samples from donors (n = 6) corresponding to different brain regions described in (http://casestudies.brain-map.org/ggb) were downloaded. A pairwise DE analysis comparing our data with the different regions of the brain was made for each donor separately using limma^30^ in R. Only brain regions having a minimum of three samples in each donor were considered for the analysis. To create a gene set collection we defined gene sets as all the pairwise contrasts made between brain regions grouping upregulated genes and downregulated genes in different gene sets. Lists of differentially expressed genes for each donor were obtained using a cut-off FDR-adjusted p-value < 0.01 together with FC > + 2 for positive enriched genes and FC < −2 for negative enriched genes. For each contrast between brain regions, only genes that appear in all the donors as DE were included in the gene set. For contrasts with a high number of DE genes, a maximum number of 800 genes were included in the gene set, considering those with lower p-value scores.

The GSEA dataset generated during this study is included as a supplementary information file. All other datasets generated during and/or analyzed during the current study are available from the corresponding author on reasonable request.

## Supplementary information

Supplementary Figures.

Supplementary information: GSEA dataset .

Supplementary Table S1.

Supplementary information .
