# Supplementary material for: Transcriptomic differences in MSA clinical variants
Source: Sci Rep. 2020 Jun 25;10:10310. doi: 10.1038/s41598-020-66221-4 (PMC7316739; doi:10.1038/s41598-020-66221-4)
Supplement: Supplementary file 1 — Supplementary Figures. [file 41598_2020_66221_MOESM1_ESM.pdf]

Transcriptomic differences in MSA clinical variants

Alexandra Pérez Soriano[1,2,3], Magdalena Arnal Segura[3, 4], Teresa Botta-Orfila[3,5], Darly Giraldo[1], Manel Fernández[2,6], Yaroslau Compta[1], Rubén Fernández-Santiago[2]*, Mario Ezquerra[2]*, Gian G. Tartaglia[3,6]*, M.J Martí[1]*, on behalf of the Catalan MSA Registry (CMSAR).

1 Parkinson's Disease & Movement Disorders Unit, Hospital Clínic / IDIBAPS / CIBERNED / European Reference Network for Rare Neurological Diseases (ERN-RND) / Institut de Neurociències, University of Barcelona, Catalonia, Spain. 2 Laboratory of Parkinson Disease and Other Neurodegenerative Movement Disorders, IDIBAPS, Barcelona, Catalonia, Spain. 3 Gene Function and Evolution Group, Centre for Genomic Regulation (CRG), Parc de Recerca Biomédica de Barcelona (PRBB), Barcelona, Catalonia, Spain. 4 Human Computational Biology Group, Hospital del Mar Medical Research Institute (IMIM), Parc de Recerca Biomédica de Barcelona (PRBB), Barcelona, Catalonia, Spain. 5 Biological Fluids Biobank; IDIBAPS-Hospital Clinic of Barcelona, Barcelona, Catalonia, Spain. 6 María de Maeztu Unit of Excellence (Institute of Neurosciences, University of Barcelona), Ministry of Science, Innovation and Universities, Barcelona, Catalonia, Spain. 7 Institucio Catalana de Recerca I Estudis Avançats (ICREA), Universitat Pompeu Fabra (UPF), Barcelona, Spain.

Corresponding author:
Prof. María José Martí, MD
Parkinson's Disease & Movement Disorders Unit
Neurology Service
Hospital Clínic de Barcelona
Carrer de Villarroel, 170
ES-08036 Barcelona
mjmarti@clinic.cat

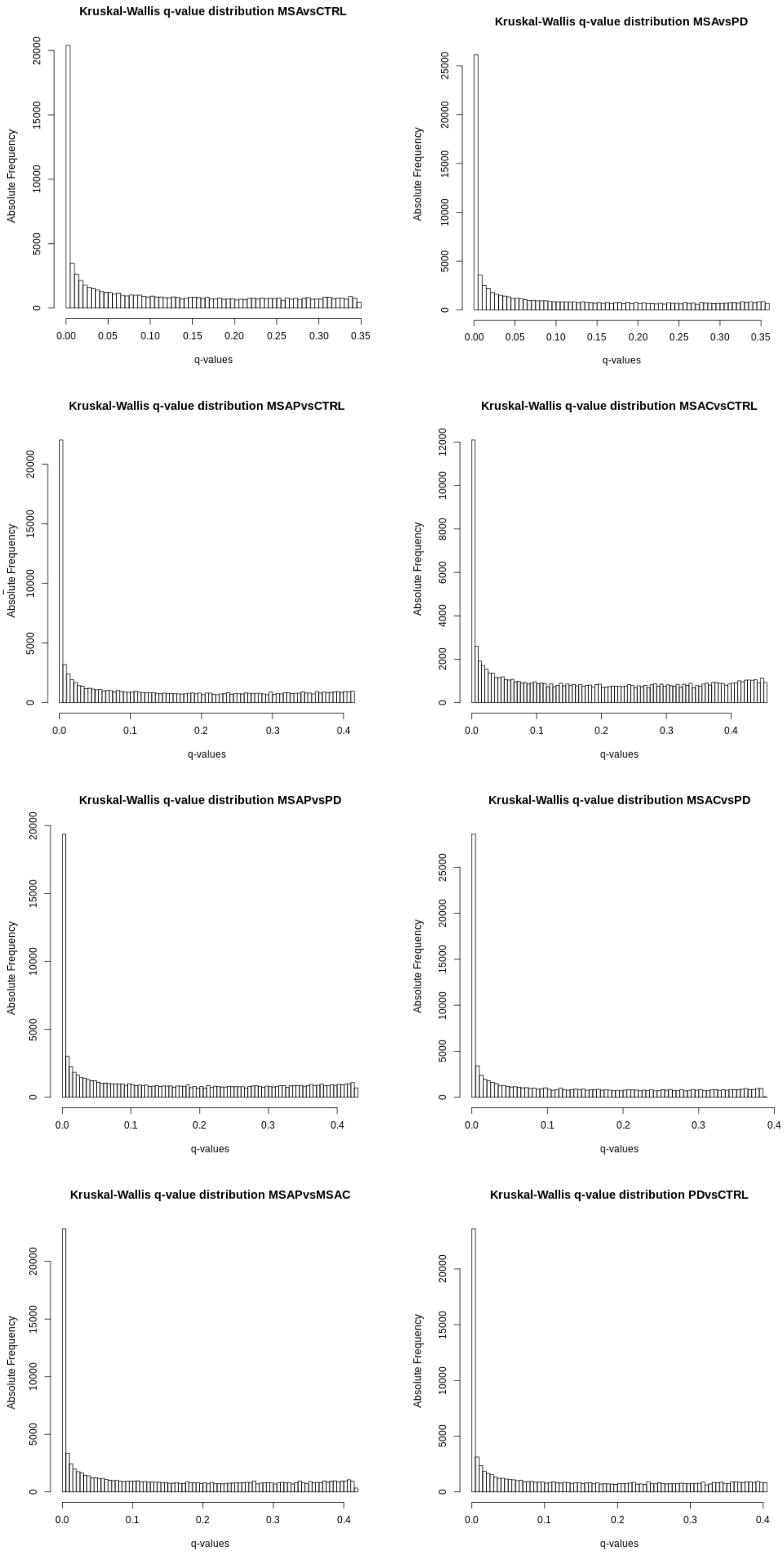

Kruskal-Wallis q-value distribution MSAvsCTRL
Absolute Frequency
q-values
Kruskal-Wallis q-value distribution MSAvsPD
Absolute Frequency
q-values
Kruskal-Wallis q-value distribution MSAPvsCTRL
Absolute Frequency
q-values
Kruskal-Wallis q-value distribution MSACvsCTRL
Absolute Frequency
q-values
Kruskal-Wallis q-value distribution MSAPvsPD
Absolute Frequency
q-values
Kruskal-Wallis q-value distribution MSACvsPD
Absolute Frequency
q-values
Kruskal-Wallis q-value distribution MSAPvsMSAC
Absolute Frequency
q-values
Kruskal-Wallis q-value distribution PDvsCTRL
Absolute Frequency
q-values

Figure S1

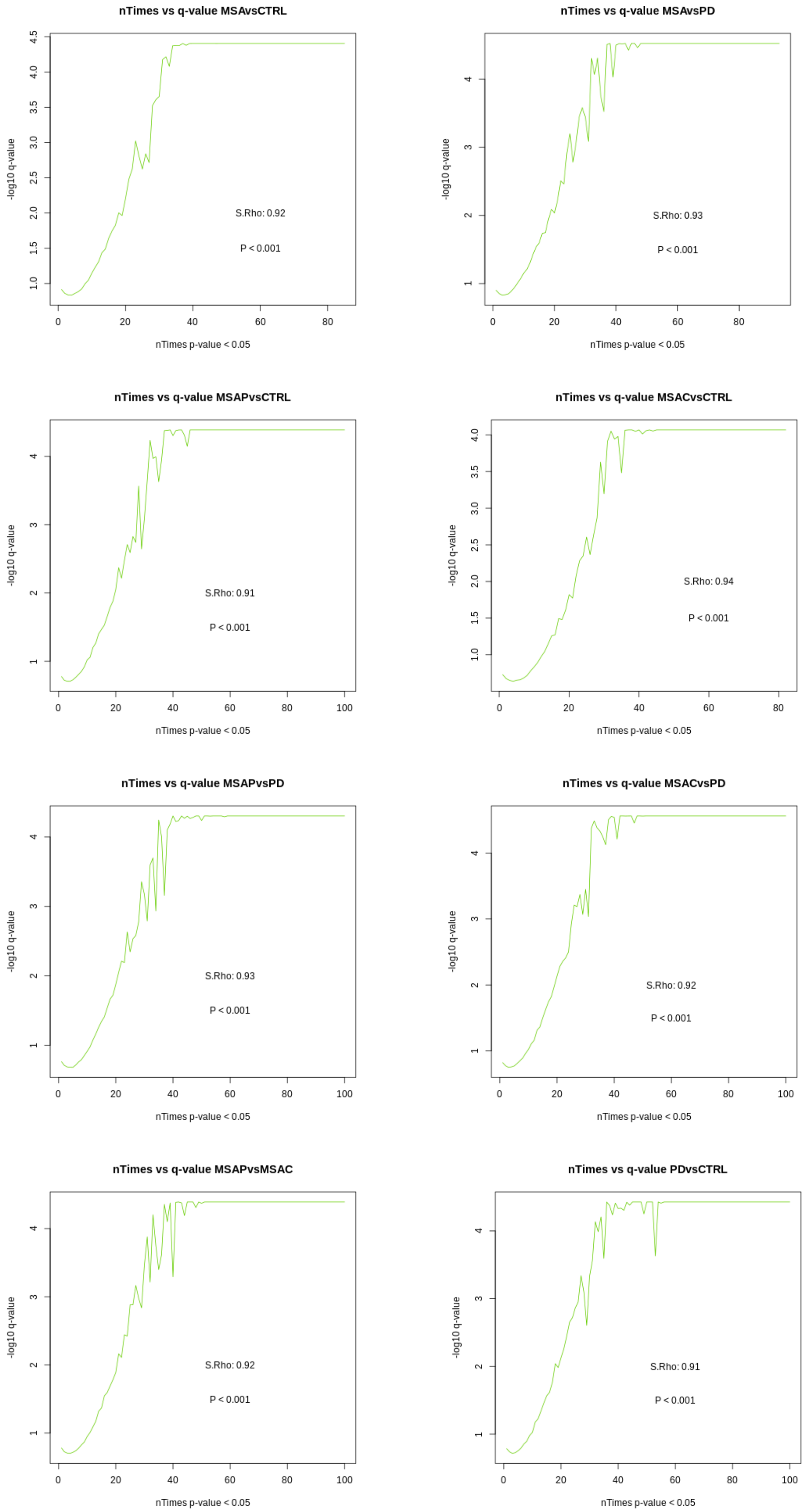
nTimes vs q-value MSAvsCTRL
-log10 q-value
nTimes p-value < 0.05
S.Rho: 0.92
P < 0.001
nTimes vs q-value MSAvsPD
-log10 q-value
nTimes p-value < 0.05
S.Rho: 0.93
P < 0.001
nTimes vs q-value MSAPvsCTRL
-log10 q-value
nTimes p-value < 0.05
S.Rho: 0.91
P < 0.001
nTimes vs q-value MSACvsCTRL
-log10 q-value
nTimes p-value < 0.05
S.Rho: 0.94
P < 0.001
nTimes vs q-value MSAPvsPD
-log10 q-value
nTimes p-value < 0.05
S.Rho: 0.93
P < 0.001
nTimes vs q-value MSACvsPD
-log10 q-value
nTimes p-value < 0.05
S.Rho: 0.92
P < 0.001
nTimes vs q-value MSAPvsMSAC
-log10 q-value
nTimes p-value < 0.05
S.Rho: 0.92
P < 0.001
nTimes vs q-value PDvsCTRL
-log10 q-value
nTimes p-value < 0.05
S.Rho: 0.91
P < 0.001

Figure S2
