# Supplementary material for: Transcriptomic differences in MSA clinical variants
Source: Sci Rep. 2020 Jun 25;10:10310. doi: 10.1038/s41598-020-66221-4 (PMC7316739; doi:10.1038/s41598-020-66221-4)
Supplement: Supplementary file 4 — Supplementary information . [file 41598_2020_66221_MOESM4_ESM.docx]

**Supplementary Figure S1: Histograms showing Kruskal-Wallis q-value distribution in the resampling analysis.**

Kruskal-Wallis q-value distribution for TCI being DE at least one time in the 100 iterations of the resampling analysis is represented in histograms with absolute frequencies for each contrast. This graph shows a peak on the left side of the histogram for all contrasts, corresponding to the statistically significant DE TCI in the resampling analysis. In case of statistically non-significant results, a homogeneous horizontal line would be seen in these plots, corresponding to the random p-value distribution in a multiple-testing approach. CTRL=control, PD=Parkinson disease, MSA=Multiple system atrophy (P=Parkinsonian phenotype, C=Cerebellar phenotype)

**Supplementary Figure S2: Correlation of Kruskal-Wallis q-values with number of times a TCI is differentially expressed in the resampling analysis.**

A Spearman correlation was applied using the vector of n times a TCI is DE in the resampling analysis and the mean of Kruskal-Wallis q-values of these DE TCI converted to the -log10 scale. Spearman Rho (S.Rho) and p-value (P) correlation statistics provided in the plots demonstrate positive correlation in all contrasts showing that most recurrent DE transcripts are also those with lower Kruskal-Wallis q-values in the resampling analysis. CTRL=control, PD=Parkinson disease, MSA=Multiple system atrophy (P=Parkinsonian phenotype, C=Cerebellar phenotype)

**Supplementary file1** (**Sfile1**): **Gene set enrichment analysis data of all contrasts.**

Top 100 significant gene sets are displayed. Gene sets are ordered by biological process group.

**Table S1: Selected TCI in the resampling analysis**

Lists of differentially expressed (DE) transcripts in the resampling analysis selected using a Kruskal-Wallis q-value < 0.0001, along with gene annotation and resampling statisticsCTRL=control, PD=Parkinson disease, MSA=Multiple system atrophy (P=Parkinsonian phenotype, C=Cerebellar phenotype). Columns in tables: AffyID=Affymetrix Transcript cluster ID (TCI), nTimes=Number of times a transcript is DE in the resampling analysis, nPos=Number of times a transcript is positive enriched, nNeg=Number of times a transcript is negative enriched, Symbol= Gene Symbol corresponding to the TCI, Krus.pVal=p-value in the Kruskal-Wallis test, Krus.qVal=q-value in the Kruskal-Wallis test.

**Table S2: Resampling analysis data.**

Summary of identified differentially expressed transcripts cluster ID (DE TCI) detected across comparisons in the resampling analysis (RA) in at least 70 of the 100 iterations (first column) with corresponding number of protein coding genes (PCG), and the number of DE TCI found in 80 of the 100 iterations (third column). Footnote: (*) Indicates a significant amount of these genes were related to RNA binding proteins by gene ontology molecular processes.

|  | **Resampling Analysis ≥ 70%** | **Protein Coding Genes** | **Resampling Analysis ≥ 80%** |
| --- | --- | --- | --- |
| MSA vs. CONTROL | 28 | 7 | 6 |
| PD vs. CONTROL | 82 | 37 | 26 |
| MSA-P vs. CONTROL | 113 | 51 | 20 |
| MSA-C vs. CONTROL | 8 | 2 | 1 |
| MSA vs. PD | 154 | 40 | 20 |
| MSA-P vs. PD | 83 | 40 | 21 |
| MSA-C vs. PD | 190 | 114* | 57 |
| MSA-P vs. MSA-C | 85 | 23 | 30 |

**Table S3: Demographic and clinical case data.**

Demographic data including gender (Men:Women), mean and standard deviation (SD) age in years, disease duration in months, Hoehn & Yahr staging (HY), Unified Multiple system atrophy rating scale (UMSARS) or Unified Parkinson’s disease rating scale (UPDRS) when applicable, presence of parkinsonism, and levodopa equivalent daily dose (LEDD) . NA=not applicable; SD=standard deviation.

| **Diagnosis** | **Gender**  **M:F** | **Age (y)** | **Disease Duration (m)** | **H&Y** | **UMSARS**  **/ UPDRS** | **Parkinsonism** | **LEDD (mg)** | **Cerebellar symptoms** |
| --- | --- | --- | --- | --- | --- | --- | --- | --- |
| MSA-P | 5:5 | 68 ± 3.8 | 68.5 ± 29.8 | 4.2 ± 0.6 | 66.1 ± 14.5 | Yes | 558 ± 168 | No |
| MSA-C | 5:5 | 66.2 ± 4.0 | 74.4 ± 40 | 3.5 ± 1.2 | 55.3 ± 18.6 | Yes | 478 ± 274 | Yes |
| PD | 5:5 | 68.5 ± 3.6 | 97.5 ± 15.8 | 1.8 ± 0.6 | 23 ± 17 | Yes | 513 ± 311 | No |
| CONTROLS | 5:5 | 67 ± 3.2 | NA | NA | NA | No | 0 | No |
